# “Benifuuki” Extract Reduces Serum Levels of Lectin-Like Oxidized Low-Density Lipoprotein Receptor-1 Ligands Containing Apolipoprotein B: A Double-Blind Placebo-Controlled Randomized Trial

**DOI:** 10.3390/nu10070924

**Published:** 2018-07-19

**Authors:** Masahiro Miyawaki, Hiroyuki Sano, Hisashi Imbe, Reiko Fujisawa, Keiji Tanimoto, Jungo Terasaki, Mari Maeda-Yamamoto, Hirofumi Tachibana, Toshiaki Hanafusa, Akihisa Imagawa

**Affiliations:** 1 Department of Internal Medicine (I), Osaka Medical College, 2-7 Daigaku-machi, Takatsuki 569-8686, Japan; in1295@osaka-med.ac.jp (M.M.); in1209@osaka-med.ac.jp (H.S.); in1297@osaka-med.ac.jp (H.I.); in1337@osaka-med.ac.jp (R.F.); in1120@osaka-med.ac.jp (K.T.); terasaki@osaka-med.ac.jp (J.T.); imagawa@osaka-med.ac.jp (A.I.); 2 National Agriculture and Food Research Organization, Agri-Food Business Innovation Center, 3-1-1 Kannondai, Tsukuba 305-8517, Japan; marimy@affrc.go.jp; 3 Division of Applied Biological Chemistry, Department of Bioscience and Biotechnology, Faculty of Agriculture, Kyushu University, 6-10-1 Hakozaki, Higashi-ku, Fukuoka 812-8581, Japan; tatibana@agr.kyushu-u.ac.jp; 4 Sakai City Medical Center, 1-1-1 Ebaraji-cho, Nishi-ku, Sakai 593-8304, Japan

**Keywords:** dyslipidemia, Benifuuki, *O*-methylated catechin, Lectin-like oxidized LDL receptor-1, LOX-1 ligand

## Abstract

(1) Background: Arteriosclerosis is associated with high levels of low-density lipoprotein (LDL) cholesterol. *O*-methylated catechins in “Benifuuki” green tea are expected to reduce cholesterol levels, although there is limited research regarding this topic; (2) Methods: This trial evaluated 159 healthy volunteers who were randomized to receive ice cream containing a high-dose of “Benifuuki” extract including 676 mg of catechins (group H), a low-dose of “Benifuuki” extract including 322 mg of catechins (group L), or no “Benifuuki” extract (group C). Each group consumed ice cream (with or without extract) daily for 12 weeks, and their lipid-related parameters were compared; (3) Results: A significant reduction in the level of lectin-like oxidized LDL receptor-1 ligand containing ApoB (LAB) was detected in group H, compared to groups L and C. No significant differences between the three groups were detected in their levels of total cholesterol, triglycerides, and LDL cholesterol; (4) Conclusions: “Benifuuki” extract containing *O*-methylated catechins may help prevent arteriosclerosis.

## 1. Introduction

Lifestyle changes, such as overeating and lack of exercise, have caused global increases in the rates of obesity, hypertension, dyslipidemia, and cardiovascular diseases [1]. As hyperlipidemia is one of the most important risk factors for cardiovascular disease, reductions in serum low-density lipoprotein (LDL) cholesterol levels can help prevent cardiovascular disease [2]. This is because oxidized LDL injures the vascular endothelium, which promotes platelet aggregation and thrombus formation. Furthermore, macrophage uptake of oxidized LDL leads to conversion into foam cells and the promotion of plaque formation, which can lead to stenosis, vascular obstruction, plaque rupture, and cardiovascular events [3,4]. Oxidized LDL is generated when LDL in the blood enters the vascular endothelium, and there is a clear relationship between increased serum levels of oxidized LDL and metabolic syndrome, such as hypertriglyceridemia [5]. Moreover, arteriosclerosis is ameliorated by reduced serum levels of oxidized LDL and the ligand, even in the presence of high levels of LDL and triglycerides [6]. Therefore, prevention of LDL oxidization, regardless of blood LDL levels, may be useful for preventing arteriosclerosis onset, progression, and cardiovascular diseases.

Green tea is a popular beverage in Japan, and previous studies have indicated that the consumption of green tea can help prevent cardiovascular events [7]. In particular, the oxidization of LDL is strongly suppressed by (−)-epigallocatechin-3-*O*-gallate (EGCG) and (−)-epicatechin-3-*O*-gallate (ECG) [8,9]. Green tea contains catechins, which are polyphenols that reduce lipase activity during the digestive process and prevent adipose micellization. These mechanisms lead to decreases in the serum levels of cholesterol and triglycerides [10,11]. Therefore, green tea catechins may reduce lipid levels and help prevent cardiovascular events.

The main catechins in green tea are (−)-epicatechin (EC), (−)-epigallocatechin (EGC), and EGCG, with EGCG providing the greatest reduction in serum cholesterol levels [11]. In addition, *O*-methylated catechins, such as (−)-epigallocatechin-3-*O*-(3-*O*-methyl)-gallate (EGCG3”Me) and (−)-gallocatechin-3-*O*-(3-*O*-methyl)-gallate (GCG3”Me), are contained in specific types of green tea (e.g., “Benifuuki” green tea), and not contained in standard green tea (e.g., “Yabukita” green tea that is most commonly consumed in Japan). These *O*-methylated catechins may have greater physiological activity than EGCG, because their high blood concentration is maintained by relatively good absorption through the gastrointestinal tract, compared to EGCG [12]. Moreover, our previous report has indicated that the consumption of “Benifuuki” green tea containing *O*-methylated catechins was associated with significantly lower serum LDL levels, compared to standard green tea containing no *O*-methylated catechins [13]. Therefore, *O*-methylated catechins may be more useful for modulating lipid metabolism, compared to standard catechins, although there is insufficient research regarding this topic.

Although green tea is a very popular beverage in Japan, its bitterness has made it relatively unpopular in Europe and America. Therefore, we propose that green tea catechins could be included in ice cream, which would mask their bitterness and improve acceptance among non-Asian populations. This randomized controlled trial examined whether ice cream containing green tea-derived *O*-methylated catechins could affect the lipid metabolism of healthy Japanese volunteers.

## 2. Materials and Methods

### 2.1. Participants and Enrolment

This study was performed in accordance with the principles of the Declaration of Helsinki, and all participants provided their written informed consent. The randomized double-blind placebo-controlled design was approved by the ethics committees of Osaka Medical College (1285-04; approval dates: 2 September 2013 to 31 March 2016) and the Osaka Medical College Health Science Clinic (2011-CR-10; approval date: 29 June 2013). Furthermore, the trial was registered in the University Hospital Medical Information Network Clinical Trials Registry (UMIN000011901). We approached 10,009 healthy individuals to recruit them for the study, in cooperation with the Osaka Medical College Health Science Clinic and using local advertisements. All potentially eligible individuals completed a careful examination that included a medical history, physical examination, and clinical testing (including blood chemistry testing). The inclusion criteria were healthy individuals who were 20–80 years old, had serum LDL cholesterol levels of >3.10 mmol/L, and had a body mass index (BMI) of >18.5 kg/m^2^. The exclusion criteria were treatment for arrhythmia, liver disorders, chronic renal diseases, cerebrovascular disorders, rheumatism, diabetes, lipid abnormalities, a history of anemia, severe allergies to specific foods and drugs, heart failure, myocardial infarction, pregnancy or a desire to become pregnant in the near future, lactation, and other conditions that were judged by the physician to preclude inclusion (e.g., the use of dietary supplements). Based on the inclusion and exclusion criteria, a total of 159 individuals were considered eligible for inclusion (56 men and 103 women who were 23–80 years old). The mean age was 53.7 ± 10.4 years and the median age was 53 years. All participants were recruited between late August of 2014 and September of the year. All participants lived in Osaka, Kyoto, or Hyogo (Japan), and underwent baseline evaluations between September 2014 and December of the year. The participants were followed for 12 weeks after the baseline evaluation.

### 2.2. Randomization

The participants were randomized 1:1:1 using a computer-generated randomization sequence, with the block size kept constant. The groups were assigned non-specific identifiers, which corresponded to group H, high-dose “Benifuuki” extract containing 676 mg of catechins (314 mg of epigallocatechin gallate (EGCG) and 66 mg of *O*-methylated catechins) and 66 mg of caffeine in ice cream; group L, low-dose “Benifuuki” extract containing 322 mg of catechins (138 mg of EGCG, 32 mg of *O*-methylated catechins) and 33 mg of caffeine in ice cream; and group C, no “Benifuuki” extract in ice cream. All participants received individually wrapped packages of ice cream with or without the extract, which were provided by an unrelated third party and were only labelled with the corresponding group identifier. Thus, the investigators and participants were blinded to the group assignments and amounts of extract in the ice cream packages.

### 2.3. Sample Size

Previous studies have evaluated 22–240 participants (group sizes of 22–120 participants) to determine whether daily consumption of catechins was associated with significant reductions in LDL cholesterol and oxidized LDL cholesterol [14,15,16]. Based on the results of those studies, we set the target sample size to 150 participants.

### 2.4. Restrictions during the Study Period

The participants were instructed to avoid excessive overeating and overdrinking starting at 1 week before the baseline evaluation, and not to modify their lifestyle (e.g., dietary habits, smoking, and exercise). Furthermore, the participants were instructed not to consume unregulated drugs and supplements that could influence their serum lipid levels. Moreover, the participants were instructed to fast starting at 10 PM on the nights before the study measurements, although they were allowed to drink water during the fasting period. Finally, the participants were instructed to refrain from smoking and extreme exercise until completion of the study, and to record their physical condition and compliance eating the ice cream (daily life record).

A limitation of our study is that polyphenols contained in chocolate, wine, and certain other foods were not evaluated in the Food Frequency Questionnaire because only the consumption of polyphenols present in tea and coffee were restricted.

### 2.5. Outcomes and Measures

The primary outcomes were defined as serum levels of total cholesterol, LDL cholesterol, high-density lipoprotein (HDL) cholesterol, and triglycerides. In addition, we evaluated lipid metabolism based on the lectin-like oxidized LDL receptor-1 index (LOX index), which is calculated by multiplying the LOX-1 ligand including ApoB (LAB) concentration by the soluble LOX-1 concentration. LAB concentration was measured using sandwich enzyme-linked immunosorbent assay. The secondary outcomes were defined as (1) body weight, waist circumference, and blood pressure; (2) fasting plasma glucose, glycated hemoglobin (HbA1c), glycoalbumin (GA), and insulin (IRI) levels; (3) pentosidine and urinary 8-hydroxydeoxyguanosine levels; (4) adiponectin levels; (5) aspartate transaminase, alanine transaminase, and γ-glutamyltransferase levels; (6) serum iron and ferritin levels; (7) urinalysis results; and (8) dietary survey responses.

**Figure 1:**
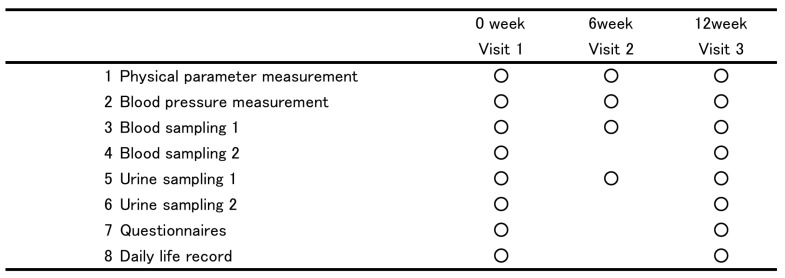
The schematic representation of study protocol. 1. the measure of height, body weight, waist circumference. 2. the measure of systolic blood pressure, diastolic blood pressure, heart rate. 3. total cholesterol, triglyceride, low-density lipoprotein cholesterol, high-density lipoprotein cholesterol, fasting plasma glucose, glycated hemoglobin (HbA1c), glycoalbumin (GA), immunoreactive insulin (IRI), aspartate transaminase (AST), alanine transaminase (ALT) and γ-glutamyltransferase (γ-GT), blood cell count, hemoglobin, hematocrit, platelet. 4. soluble lectin-like oxidized LDL receptor-1 (sLOX-1), LOX-1 ligand including ApoB(LAB), LOX index, pentosidine, adiponectin, serum iron and ferritin. 5. Urinalysis. 6. urinary 8-hydroxydeoxyguanosine (8-OH-dG) level. 7. the Food Frequency Questionnaire. 8. records of their individual adherence to eating test ice cream and their physical conditions.

The participants’ body weights were measured using bioelectricity impedance analysis (InnerScan^®^50V BC-621-SS; Tanita Corporation, Tokyo, Japan). Blood pressure was measured twice, before and after a 3-min rest, using a sphygmomanometer (HEM-7200^®^; Omron Corporation, Kyoto, Japan). Biochemical tests were performed by SRL Inc. (Tokyo, Japan). The participants also completed questionnaires regarding their health, lifestyle, food intake, type of green tea that they consumed, and the frequency of green tea consumption. Intake of tea catechins was evaluated using the food frequency questionnaire from the Osaki National Health Insurance cohort study [7]. We show the study’s protocol in Figure 1.

### 2.6. Ice Cream and Intervention

Ice cream packages (125 mL (110 g), 138 kcal/package) with no extract, as well as packages with high or low levels of “Benifuuki” extract, were manufactured by MORINAGA & Co., Ltd. (Tokyo, Japan). The nutritional profile and the content of catechins and caffeine of the test ice cream is shown in Table 1. The analysis of catechins and caffeine in “Benifuuki” extract was performed as previously described [17]. All participants received packages of ice cream that corresponded to their blinded group assignment and were instructed to consume one package per day for 12 weeks.

### 2.7. Statistical Methods

Differences between groups H, L, and C were evaluated based on the intention-to-treat principle and using the Kruskal-Wallis test, the Steel–Dwass test, the Steel test, and the paired *t*-test, as appropriate. All analyses were performed using JMP software (version 11.2.1; SAS Research Institute Corporation, Carey, NC, USA). Differences were considered statistically significant at a *p*-value of <0.05.

## 3. Results

### 3.1. Participants and Follow-Up

**Figure 2:**
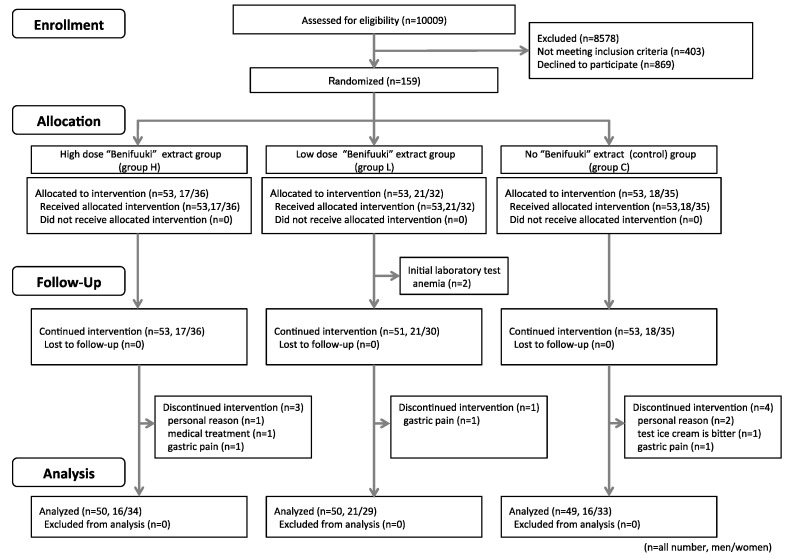
Flowchart of the study.

The 159 participants were allocated to group H (*n* = 53, 17 men and 36 women), group L (*n* = 53, 21 men and 32 women), and group C (*n* = 53, 18 men and 35 women), although ten participants were subsequently excluded from the analysis. At the baseline evaluation, two participants from group L were disqualified because they had developed anemia. During the study period, three participants in group H were excluded (withdrawal for personal reasons, gastric pain, and treatment of a previous illness). One participant in group L was excluded because of gastric pain. One participant in group C was disqualified because they could not tolerate the daily ice cream consumption, two participants withdrew because of personal reasons, and one participant withdrew because of gastric pain. Thus, 149 participants (53 men and 96 women) completed the study, with 50 participants in group H (16 men and 34 women), 50 participants in group L (21 men and 29 women), and 49 participants in group C (16 men and 33 women). We show the flowchart of the study in Figure 2.

### 3.2. Baseline Data and Outcomes

**Figure 3:**
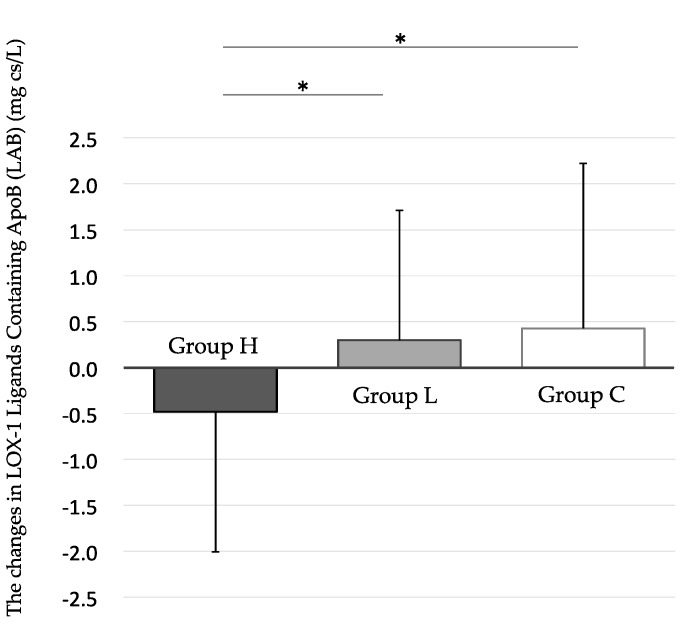
The changes in LOX-1 ligands containing ApoB (LAB) level from 0 to 12 weeks. The Steel–Dwass test shows significant differences in the Δ value at 12 weeks between group H and group L, and between group H and group C. Data are shown as mean ± SD, * *p* < 0.05. Black bar: High-dose “Benifuuki” extract ice cream group (group H). Gray bar: Low-dose “Benifuuki” extract ice cream group (group L). White bar: No “Benifuuki” extract ice cream (control) group (group C).

The overall compliance rate for the ice cream consumption was 96.0 ± 6.1%, and there was no significant inter-group difference in the compliance rates (group H, 96.2 ± 5.5%; group L, 94.5 ± 7.8%; group C, 97.5 ± 4.2%). No significant differences were observed between the three groups in their baseline values for weight, BMI, abdominal circumference, and γ-glutamyltransferase levels. By the paired *t* test, a significant decrease in group H’s LAB level was detected at the 12-week follow-up, compared to the baseline value in that group. However, no meaningful reductions were observed in the week 12 values for LAB in groups L and C, compared to their respective baseline values. And, by the Steel–Dwass test, a significant reduction in the level of LAB was detected in group H, compared to both groups L and C, at the 12-week follow-up (Figure 3) (Table 2).

No significant inter-group differences were observed in the levels of total cholesterol and LDL cholesterol when we compared the baseline and week 12 values (Table 2).

In addition, the Steel–Dwass test showed significant differences in the Δ value of GA at 12 weeks between group H and group L, and between group H and group C, but showed no significant differences in the Δ value of HbA1c (Table 2).

Furthermore, no significant differences were observed in the participants’ questionnaire responses and life records. Moreover, based on the food frequency questionnaire responses, no significant differences were observed in the participants’ daily caloric intakes when we compared the baseline and week 12 values.

### 3.3. Ancillary Analysis

A subgroup analysis was performed between the two participant groups with (*n* = 105) and without (*n* = 44) a habit of daily tea drinking. In the both groups, no endpoints had any significant change, suggesting that the division of the study population weakened the detection power.

On the other hand, the differences adjusted for age and sex between groups H, L, and C were evaluated using the analyses of covariance (ANCOVA) test. The analyses adjusted for age and sex showed significant differences in the Δ value of LAB level at 12 weeks between three groups. We show the result of the analyses in Table 3.

### 3.4. Adverse Events

None of the participants reported experiencing adverse events that could be attributed to the ice cream or catechins, although a previous report has indicated that green tea catechin consumption can be associated with liver damage or low levels of iron [18].

## 4. Discussion

The present study revealed that consumption of “Benifuuki” extract in ice cream was associated with a significant reduction in LAB levels. The LAB levels reflect various types of modification (e.g., oxidization, malondialdehyde modification, acetylation, carbamylation, and glycation) of LDL [19]. Progression of arteriosclerosis is driven by the binding of modified LDL to LOX-1 in the vascular endothelium. This promotes endothelial injury through apoptosis, release of inflammatory cytokines, production of active oxygen, and reduction of nitric oxide levels [20]. In addition, LOX-1 is expressed in smooth muscle cells, macrophages, and platelets, where it can cause smooth muscle cell proliferation, foam cell formation, and aggregation of activated platelets [21]. In this context, LAB levels are suggested to be a new biomarker for evaluating the progression of arteriosclerosis and related diseases. For example, recent studies have suggested that LAB and the carotid intima-media thickness were significantly and positively correlated [22,23]. In addition, a recent study reported that LAB showed significant positive correlations with history of smoking, waist circumference, triglycerides [24]. In the present study, the Δ value of LAB at 12 weeks showed significant positive correlations with the Δ value of body weight, the Δ value of BMI, and the Δ value of triglyceride by the Spearman’s rank correlation coefficient test. However, the Δ values of body weight and BMI at 12 weeks were not significantly different between the three groups, suggesting that no significant correlations exist between the Δ value of LAB and the Δ value of body weight and BMI. Significantly positive correlations between the Δ value of triglyceride and the Δ value of LAB at 12 weeks were detected only in group C, and was not detected either in group H or L (the data shown in Supplementary material Table S1). However, the Δ value of triglyceride at 12 weeks had no significant difference between the three groups, suggesting that a significant decrease in group H’s LAB level at the 12 weeks was not related with triglyceride.

On the other hand, consumption of “Benifuuki” extract in ice cream was not associated with a reduction in LDL cholesterol levels. One possible explanation is that significant changes in cholesterol levels were not clear, in the participants without lipid abnormalities and without overweight. Alternatively, the doses of catechin may have been insufficient to affect cholesterol levels in such participants. Finally, all of the participants had a pre-existing habit of drinking green tea, which might have obscured any effects of the catechins in the ice cream.

In addition, by the result of this study, the Steel–Dwass test showed significant differences in the Δ value of GA at 12 weeks between group H and group L, and between group H and group C, but showed no significant differences in the Δ value of HbA1c. These results suggested that high-dose “Benifuuki” extract might have some influences on the participants’ glycemic control, compared to both low-dose “Benifuuki” extract and Control. However, the influence could be too minimal or short-lasting to significantly change HbA1c level, and may have no clinical significance, because GA is a useful clinical biomarker for glycemic control and reflects a short-term glycemic control, compared to HbA1c, which reflects long-term glycemia.

## 5. Conclusions

In the present study, daily consumption of “Benifuuki” extract (676 mg of catechins per day) was associated with a significant decrease in serum LAB levels, compared to the group that received less extract (322 mg of catechins per day) or no extract. We attribute this effect to the “Benifuuki” extract containing *O*-methylated catechins, which may help suppress the progression of arteriosclerosis.

## Figures and Tables

**Table 1:** Nutritional profile and catechins and caffeine content of the test ice cream provided per day.

	Placebo	Benifuuki Extract Low-Dose	Benfuuki Extract High-Dose
calories (kcal)	138	138	138
protein (g)	3.3	3.3	3.3
fat (g)	6.9	6.9	6.9
carbohydrate (g)	21.1	20.1	18.8
Sodium choloride (mg)	58	56	56
total catechins (mg)	0	322	676
total *O*-methyltated catechins ((−)-epigallocatechin-3-*O*-(3-*O*-methyl) gallate + (−)-gallocatechin-3-*O*-(3-*O*-methyl) gallate) (mg)	0	32	66
(−)-epigallocatechin-3-*O*-gallate + (−)-gallocatechin-3-*O*-gallate (mg)	0	138	314
caffeine	0	33	66

**Table 2:** The characteristics and parameters at baseline (week 0) and week 12.

	Study Group	0 Week	12 Week	Δ Value at 12 Weeks ^a^	Paired *t* Test ^b^	Kruskal-Wallis Test ^c^	Steel–Dwass Test ^d^	Steel Test ^e^
					*p*-Value	*p*-Value	*p*-Value	*p*-Value
Baseline characteristics								
Sex	H group	16/34						
(male/female)	L group	18/32						
	C group	19/30						
Age	H group	52.1 ± 10.9						
(year)	L group	54.4 ± 9.7						
	C group	53.8 ± 11.2						
Diet survey								
Total daily intake	H group	2109.0 ± 1055.6	2160.0 ± 1178	51.0 ± 835.1	0.6676	0.4977	§: 0.7108	§: 0.6455
(kcal/day)	L group	2009.2 ± 539.7	2128.6 ± 946.1	119.3 ± 802.3	0.8101		§§: 0.7322	§§: 0.6696
	C group	2215.1 ± 903.9	2175.5 ± 902.6	−39.5 ± 1145.1	0.2980		§§§: 0.9759	
Anthropometric values								
Body weight	H group	65.5 ± 10.6	66.2 ± 10.7	0.7 ± 1.3	0.0003 ***	0.8074	§: 0.6455	§: 0.9777
(kg)	L group	60.9 ± 8.5	61.5 ± 8.6	0.6 ± 1.1	<0.0001 ****		§§: 0.6696	§§: 0.6695
	C group	62.0 ± 8.9	62.8 ± 9.0	0.8 ± 1.1	<0.0001 ****		§§§: 0.6696	
Body Mass Index	H group	24.9 ± 3.6	25.1 ± 3.7	0.3 ± 0.5	0.0002 ***	0.8112	§: 0.9618	§: 0.9494
(kg/m^2^)	L group	23.5 ± 2.5	23.7 ± 2.5	0.2 ± 0.4	<0.0001 ****		§§: 0.7615	§§: 0.7032
	C group	23.5 ± 2.6	23.9 ± 2.5	0.3 ± 0.4	<0.0001 ****		§§§: 0.9666	
Waist circumference	H group	90.0 ± 8.5	89.2 ± 9.1	−0.8 ± 2.1	0.0082 **	0.9879	§: 0.9989	§: 0.9985
(cm)	L group	85.9 ± 6.8	85.2 ± 6.9	−0.7 ± 2.2	0.0328 *		§§: 0.9882	§§: 0.9842
	C group	87.1 ± 7.2	86.4 ± 7.1	−0.7 ± 2.2	0.0353 *		§§§: 0.9929	
SBP	H group	122.0 ± 15.6	122.6 ± 13.8	0.7 ± 11.2	0.6687	0.4400	§: 0.6998	§: 0.6333
(mmHg)	L group	127.5 ± 19.6	126.9 ± 18.5	−0.7 ± 11.5	0.6901		§§: 0.8209	§§: 0.7731
	C group	120.6 ± 14.0	119.2 ± 15.7	−1.3 ± 13.4	0.4991		§§§: 0.4532	
DBP	H group	75.5 ± 10.1	76.9 ± 9.1	1.4 ± 6.3	0.1312	0.5862	§: 0.5870	§: 0.5118
(mmHg)	L group	78.4 ± 10.6	79.2 ± 10.0	0.8 ± 5.0	0.2731		§§: 0.9996	§§: 0.9994
	C group	75.1 ± 9.1	75.4 ± 8.6	0.4 ± 5.4	0.6465		§§§: 0.7092	
Pulse	H group	70.1 ± 12.0	71.8 ± 11.6	1.8 ± 8.1	0.1311	0.2087	§: 0.3121	§: 0.2484
(b.p.m.)	L group	72.0 ± 8.9	70.8 ± 8.9	−1.9 ± 9.6	0.3122		§§: 0.9908	§§: 0.9877
	C group	72.1 ± 11.8	70.2 ± 10.3	−1.2 ± 8.4	0.1796		§§§: 0.2516	
Lipid parameters								
Total cholesterol	H group	236.2 ± 26.5	246.5 ± 26.3	10.3 ± 36.2	0.0493 *	0.3176	§: 0.8395	§: 0.7955
(mg/dL)	L group	247.1 ± 30.0	248.6 ± 27.7	1.5 ± 42.0	0.7992		§§: 0.6007	§§: 0.5261
	C group	236.7 ± 24.2	245.2 ± 27.7	8.6 ± 32.7	0.0734		§§§: 0.3061	
Triglyceride	H group	112.9 ± 51.2	103.0 ± 48.2	−9.9 ± 41.6	0.0981	0.3753	§: 0.8152	§: 0.7663
(mg/dL)	L group	116.1 ± 60.6	112.3 ± 48.7	−3.8 ± 42.8	0.5289		§§: 0.8711	§§: 0.8342
	C group	105.8 ± 51.4	103.1 ± 54.3	−2.7 ± 33.9	0.5886		§§§: 0.2781	
HDL cholesterol	H group	58.0 ± 13.5	62.6 ± 15.3	4.6 ± 6.8	<0.0001 ****	0.1637	§: 0.6373	§: 0.5649
(mg/dL)	L group	61.8 ± 13.5	66.5 ± 14.3	4.7 ± 5.6	<0.0001 ****		§§: 0.1486	§§: 0.1113
	C group	59.7 ± 12.8	65.7 ± 13.9	6.0 ± 6.0	<0.0001 ****		§§§: 0.5472	
LDL choresterol	H group	155.6 ± 25.9	163.3 ± 29.3	7.7 ± 36.1	0.1399	0.5772	§: 0.6604	§: 0.6503
(mg/dL)	L group	162.1 ± 26.2	159.7 ± 30.7	−2.4 ± 42.4	0.6870		§§: 0.6183	§§: 0.6960
	C group	155.8 ± 20.2	158.9 ± 28.6	3.1 ± 32.2	0.5030		§§§: 0.9862	
LOX index-associated parameters								
sLOX-1	H group	271.8 ± 135.8	232.5 ± 101.4	−39.3 ± 110.1	0.0150 *	0.4188	§: 0.4656	§: 0.3901
(ng/L)	L group	297.3 ± 137.7	231.3 ± 94.8	−65.8 ± 103.7	<0.0001 ****		§§: 0.9839	§§: 0.9786
	C group	285.2 ± 181.3	207.6 ± 76.6	−77.5 ± 161.2	0.0015 **		§§§: 0.5272	
LAB	H group	6.2 ± 2,2	5.7 ± 2.5	−0.5 ± 1.5	0.0296 *	0.0120 *	§: 0.0337 *	§: 0.0238 *
(mg cs/L)	L group	6.1 ± 2.7	6.4 ± 3.4	0.3 ± 1.4	0.1421		§§: 0.9983	§§: 0.9977
	C group	6.3 ± 2.7	6.7 ± 3.3	0.4 ± 1.8	0.1042		§§§: 0.0228 *	
LOX index	H group	1682.3 ± 994.5	1312.3 ± 764.8	−370.0 ± 624.9	0.0001 ***	0.7130	§: 0.7778	§: 0.7222
	L group	1807.1 ± 1122.9	1455.9 ± 924.4	−351.2 ± 537.2	<0.0001 ****		§§: 0.7511	§§: 0.6913
	C group	1785.6 ± 1454.1	1388.2 ± 797.9	−397.3 ± 1025.6	0.0093 **		§§§: 0.9710	
Glycometabolism-associated parameters								
HbA1c	H group	5.28 ± 0.26	5.32 ± 0.25	0.04 ± 0.03	0.0701	0.6052	§: 0.7259	§: 0.6625
(%)	L group	5.24 ± 0.25	5.31 ± 0.29	0.07 ± 0.24	0.0529		§§: 0.9890	§§: 0.9853
	C group	5.22 ± 0.29	5.30 ± 0.32	0.08 ± 0.21	0.0115 *		§§§: 0.6140	
GA	H group	14.15 ± 1.10	13.77 ± 1.13	−0.38 ± 0.33	<0.0001 ****	0.0022 *	§:0.0191 *	§:0.0133 *
(%)	L group	14.01 ± 0.85	13.82 ± 0.82	−0.19 ± 0.32	<0.0001 ****		§§: 0.7859	§§: 0.7316
	C group	14.13 ± 1.08	13.91 ± 1.11	−0.22 ± 0.31	<0.0001 ****		§§§: 0.0035 **	
Blood glucose	H group	89.9 ± 6.4	90.6 ± 9.0	0.7 ± 6.7	0.4394	0.9474	§: 0.9790	§: 0.9721
(mg/dL)	L group	89.6 ± 7.5	90.0 ± 7.2	0.4 ± 5.4	0.6405		§§: 0.9357	§§: 0.9157
	C group	89.2 ± 8.7	89.3 ± 6.9	0.4 ± 7.1	0.7037		§§§: 0.9987	
IRI	H group	6.5 ± 4.2	6.9 ± 4.2	0.4 ± 2.3	0.2674	0.2453	§: 0.5301	§: 0.4536
(μU/mL)	L group	5.1 ± 2.6	5.8 ± 3.6	0.7 ± 2.5	0.0589		§§: 0.2505	§§: 0.1951
	C group	6.3 ± 6.9	5.5 ± 2.4	−0.8 ± 6.6	0.4081		§§§: 0.7493	
HOMA-IR	H group	1.4 ± 0.9	1.6 ± 1.0	0.1 ± 0.6	0.1602	0.2848	§: 0.5213	§: 0.4449
	L group	1.1 ± 0.6	1.3 ± 0.8	0.1 ± 0.6	0.0714		§§: 0.2708	§§: 0.2124
	C group	1.5 ± 2.1	1.1 ± 0.6	−0.2 ± 2.0	0.4049		§§§: 0.9057	
Cytokines								
Adiponectin	H group	3.6 ± 2.3	3.9 ± 2.5	0.3 ± 0.7	0.0025 **	0.8585	§: 0.8859	§: 0.8525
(μg/mL)	L group	4.0 ± 2.3	3.8 ± 2.0	0.3 ± 0.5	0.0016 **		§§: 0.8875	§§: 0.8545
	C group	3.5 ± 1.9	4.3 ± 2.5	0.3 ± 0.6	0.0017 **		§§§: 0.9890	
A biomarker for advances glycation end products								
Pentosidine	H group	0.037 ± 0.010	0.045 ± 0.013	0.008 ± 0.016	0.0018 **	0.6181	§: 0.9736	§: 0.9649
(μg/mL)	L group	0.037 ± 0.009	0.046 ± 0.013	0.009 ± 0.013	<0.0001 ****		§§: 0.5763	§§: 0.5008
	C group	0.038 ± 1.000	0.044 ± 0.013	0.007 ± 0.014	0.0022 **		§§§: 0.8089	
Complete blood count								
Hb	H group	14.2 ± 1.0	14.5 ± 1.2	0.3 ± 0.6	0.0038 *	0.9913	§: 0.9913	§: 0.9883
(g/dL)	L group	14.0 ± 1.3	14.3 ± 1.5	0.3 ± 0.6	0.0019 *		§§: 0.9983	§§: 0.9977
	C group	14.1 ± 1.1	14.4 ± 1.2	0.3 ± 0.6	0.0015 *		§§§: 0.9971	
Hct	H group	42.5 ± 3.2	43.5 ± 3.5	0.9 ± 1.9	0.0015 *	0.8793	§: 0.9103	§: 0.8832
(%)	L group	41.9 ± 3.7	42.9 ± 4.2	1.0 ± 1.8	0.0002 *		§§: 0.9985	§§: 0.9980
	C group	43.1 ± 3.2	43.1 ± 3.6	1.0 ± 2.0	0.0008 *		§§§: 0.8936	
RBC	H group	459.9 ± 35.4	466.2 ± 33.5	6.3 ± 20.8	0.0364 *	0.8523	§: 0.9913	§: 0.9884
(×10^4^/μL)	L group	453.4 ± 33.7	460.3 ± 38.4	6.9 ± 18.7	0.0122 *		§§: 0.9479	§§: 0.9314
	C group	454.7 ± 37.6	462.8 ± 42.1	8.2 ± 26.0	0.0328 *		§§§: 0.8182	
Plt	H group	24.9 ± 5.0	27.9 ± 13.8	3.1 ± 12.7	0.0940	0.4660	§: 0.9931	§: 0.9908
(×10^4^/μL)	L group	25.6 ± 5.9	25.9 ± 5.9	0.3 ± 2.5	0.3404		§§: 0.4383	§§: 0.3640
	C group	25.5 ± 5.3	26.1 ± 4.6	0.6 ± 2.6	0.0921		§§§: 0.6535	
WBC	H group	5298.0 ± 1307.0	5616.0 ± 1553.8	318.0 ± 1422.8	0.1204	0.2471	§: 0.3171	§: 0.2528
(/μL)	L group	5068.0 ± 1299.1	5200 ± 1149.1	132.0 ± 1080.9	0.3921		§§: 0.3268	§§: 0.2615
	C group	5187.8 ± 1426.5	5183.7 ± 1339.4	−4.1 ± 938.5	0.9758		§§§: 0.9656	
Liver functions								
AST	H group	22.5 ± 6.7	21.7 ± 5.4	−0.9 ± 6.3	0.3378	0.8213	§: 0.9651	§: 0.9538
(U/L)	L group	24.9 ± 10.6	24.7 ± 9.5	−0.0 ± 3.2	0.8932		§§: 0.9044	§§: 0.8758
	C group	22.0 ± 5.3	22.0 ± 5.3	−0.1 ± 7.3	0.9298		§§§: 0.8312	
ALT	H group	25.4 ± 15.8	25.3 ± 13.0	−0.1 ± 11.8	0.9618	0.2705	§: 0.2441	§: 0.1897
(U/L)	L group	22.7 ± 14.8	23.1 ± 13.6	0.5 ± 10.6	0.7597		§§: 0.4827	§§: 0.4067
	C group	21.7 ± 11.5	22.0 ± 10.7	0.3 ± 4.7	0.6749		§§§: 0.9817	
γGTP	H group	30.1 ± 22.3	31.7 ± 27.2	1.6 ± 8.1	0.1653	0.4453	§: 0.3913	§: 0.3199
(U/L)	L group	41.0 ± 35.6	42.2 ± 38.3	1.2 ± 13.8	0.5484		§§: 0.6982	§§: 0.6314
	C group	23.8 ± 14.7	23.9 ± 14.9	0.1 ± 4.6	0.8516		§§§: 0.9617	
Iron metabolism								
Fe	H group	19.3 ± 6.0	18.8 ± 7.1	−0.5 ± 7.3	0.6542	0.3897	§: 0.6716	§: 0.6021
(μmol/L)	L group	18.6 ± 6.7	19.2 ± 6.5	0.6 ± 7.2	0.5488		§§: 0.3785	§§: 0.3081
	C group	19.1 ± 5.4	17.6 ± 5.7	−1.6 ± 6.2	0.0772		§§§: 0.8403	

H group: “Benifuuki” green tea extract high component ice cream group. L group: “Benifuuki” green tea extract low component ice cream group. C group: “Benifuuki” green tea extract non-component ice cream (control) group. All data are mean ± standard deviation. ^a^: The value is the change from 0 to 12 weeks. ^b^: Paired *t* test analyzed in each group to compare the data at 0 week (baseline) and 12 weeks (* *p* < 0.05, ** *p* < 0.01, *** *p* < 0.001, **** *p* < 0.0001). ^c^: Kruskal–Wallis test analyzed the statistical difference of the Δ value at 12 weeks between all groups. (* *p* < 0.05, ** *p* < 0.01, *** *p* < 0.001, **** *p* < 0.0001). ^d^: Steel–Dwass test analyzed the statistical difference of the Δ value at 12 weeks in H group. or L group compared to C group. (§: between H group and C group, §§: between L group and C group, §§§: between H group and L group, * *p* < 0.05, ** *p* < 0.01, *** *p* < 0.001, **** *p* < 0.0001). ^e^: Steel test analyzed the statistical difference of the Δ value at 12 weeks in H group or L group compared to C group. (§: between H group and C group, §§: between L group and C group, * *p* < 0.05, ** *p* < 0.01, *** *p* < 0.001, **** *p* < 0.0001). SBP: systolic blood pressure, DBP: diastolic blood pressure, sLOX-1: soluble lectin-like oxidized LDL receptor-1, LAB: LOX-1 ligand including ApoB, LOX index: lectin-like oxidized LDL receptor-1 index, HbA1c: glycated hemoglobin, GA: glycoalbumin, IRI: immunoreactive insulin, HOMA-IR: Homeostatic Model Assessment for Insulin Resistance, Hb: hemoglobin, Hct: hematocrit, RBC: red blood cell, Plt: platelet, WBC: white blood cell, AST: aspartate transaminase, ALT: alanine transaminase, γ-GT: γ-glutamyltransferase.

**Table 3:** The age- and sex-adjusted analyses in the Δ value of sLOX-1, LAB and LOX index at 12 weeks between three groups.

	Kruskal–Wallis Test ^a^	ANCOVA Test ^b^ (Adjusted for Age)	ANCOVA Test ^b^ (Adjusted for Sex)
	*p*-Value	*p*-Value	*p*-Value
the Δ value of sLOX-1 (ng/L)	0.4188	0.3432	0.2901
the Δ value of LAB (mg cs/L)	0.0120 *	0.0149 *	0.0122 *
the Δ value of LOX index	0.7130	0.9543	0.9378

^a^: Kruskal-Wallis test analyzed the statistical difference of the Δ value at 12 weeks between all groups (* *p* < 0.05). ^b^: ANCOVA test analyzed the statistical difference of the Δ value at 12 weeks between all groups (* *p* < 0.05), the Δ value: the increase or decrease level compared to the baseline value; sLOX-1: soluble lectin-like oxidized LDL receptor-1; LAB: LOX-1 ligand including ApoB; LOX index: lectin-like oxidized LDL receptor-1 index.

## Supplementary Materials

The following are available online at http://www.mdpi.com/2072-6643/10/7/924/s1, Table S1: The analysis of correlation between Lipid parameters and LAB at 12 weeks.
