# Supplementary material for: “Benifuuki” Extract Reduces Serum Levels of Lectin-Like Oxidized Low-Density Lipoprotein Receptor-1 Ligands Containing Apolipoprotein B: A Double-Blind Placebo-Controlled Randomized Trial
Source: Nutrients. 2018 Jul 19;10(7):924. doi: 10.3390/nu10070924 (PMC6073342; doi:10.3390/nu10070924)
Supplement: Supplementary file 1 [file nutrients-10-00924-s001.pdf]

Table S1. The analysis of correlation between Lipid parameters and LAB at 12 weeks.

	the Spearman's rank correlation coefficient test [a]	
	ρ-value	p-value
**between the Δ value of total cholesterol and the Δ value of LAB**		
All groups	-0.0160	0.8465
Group H	0.0536	0.7119
Group L	-0.1134	0.4329
Group C	0.1262	0.3877
**between the Δ value of triglycerides and the Δ value of LAB**		
All groups	0.1838	0.0249*
Group H	0.0972	0.5018
Group L	0.0579	0.6897
Group C	0.3442	0.0154*
**between the Δ value of HDL and the Δ value of LAB**		
All groups	0.0579	0.4828
Group H	0.0601	0.6782
Group L	0.0408	0.7783
Group C	-0.0445	0.7612
**between the Δ value of LDL and the Δ value of LAB**		
All groups	-0.1356	0.3479
Group H	0.0481	0.7400
Group L	-0.1356	0.3479
Group C	0.0828	0.5719

a : the Spearman's rank correlation coefficient test analyzed the statistical difference of the Δ value at 12 weeks.(*p<0.05, **p<0.01, ***p<0.001, ****p<0.0001) .
